# Satisfaction at work as a determinant of the mental health of electricians

**DOI:** 10.1192/j.eurpsy.2023.2024

**Published:** 2023-07-19

**Authors:** I. Sellami, A. Feki, A. Abbes, M. A. Ghrab, S. Baklouti, M. L. Masmoudi, K. Jmal Hammami, M. Hajjaji

**Affiliations:** 1 occupational medecine, Hedi Chaker Hospital, university of Sfax; 2 Rheumatology; 3 occupational medecine, University of Sfax; 4 Rheumatology, Hedi Chaker Hospital, university of Sfax, Sfax, Tunisia

## Abstract

**Introduction:**

The workplace environment influences employees’ health. Authors widely recognise job dissatisfaction as a workplace. Work satisfaction can influence employees’ psychological health statuses.

**Objectives:**

We aimed to assess the impact of satisfaction at work on the mental health of electricians.

**Methods:**

The study concerned a group of electricians who agreed to answer a face-to-face interview concerning working conditions and mental health status. Data was collected using a pre-designed questionnaire that included socio-professional characteristics, a 10-point scale of job satisfaction, and the Kessler Psychological Distress Scale (K6) between January and June 2022.

**Results:**

Our study included 74 male electricians. The mean age was 39.3 ± 10.5 years. The average job tenure was 15.5 ± 11.2 years. The mean score of K6 was 5.4±4.8 (range = 0–22). The proportion of respondents with high levels of psychological distress (K6 score of 13 or greater) was 9.5 %. The mean score of satisfaction at work was 7.7±1.8. Low satisfaction at work was correlated with high levels of psychological distress (p = 0.012; r = -0.29).

**Conclusions:**

High job satisfaction was correlated with low levels of psychological distress. Policies and practices should focus on improving working conditions to enhance the mental health of employees.

**Disclosure of Interest:**

None Declared

